# Differential Regulation of Genes for Cyclic-di-GMP Metabolism Orchestrates Adaptive Changes During Rhizosphere Colonization by *Pseudomonas fluorescens*

**DOI:** 10.3389/fmicb.2019.01089

**Published:** 2019-05-16

**Authors:** Richard H. Little, Stuart D. Woodcock, Rosaria Campilongo, Rowena K. Y. Fung, Robert Heal, Libby Humphries, Alba Pacheco-Moreno, Stefan Paulusch, Egidio Stigliano, Eleni Vikeli, Danny Ward, Jacob G. Malone

**Affiliations:** ^1^Department of Molecular Microbiology, John Innes Centre, Norwich, United Kingdom; ^2^Internal Medicine, Universitätsklinikum Bonn, Bonn, Germany; ^3^School of Biological Sciences, University of East Anglia, Norwich, United Kingdom

**Keywords:** cyclic-di-GMP, *Pseudomonas fluorescens*, diguanylate cyclase, phosphodiesterase, plant colonization

## Abstract

Bacteria belonging to the *Pseudomonas* genus are highly successful colonizers of the plant rhizosphere. The ability of different *Pseudomonas* species to live either commensal lifestyles or to act as agents of plant-growth promotion or disease is reflected in a large, highly flexible accessory genome. Nevertheless, adaptation to the plant environment involves a commonality of phenotypic outputs such as changes to motility, coupled with synthesis of nutrient uptake systems, stress-response molecules and adherence factors including exopolysaccharides. Cyclic-di-GMP (cdG) is a highly important second messenger involved in the integration of environmental signals with appropriate adaptive responses and is known to play a central role in mediating effective rhizosphere colonization. In this study, we examined the transcription of multiple, reportedly plant-upregulated cdG metabolism genes during colonization of the wheat rhizosphere by the plant-growth-promoting strain *P. fluorescens* SBW25. While transcription of the tested genes generally increased in the rhizosphere environment, we additionally observed a tightly orchestrated response to environmental cues, with a distinct transcriptional pattern seen for each gene throughout the colonization process. Extensive phenotypical analysis of deletion and overexpression strains was then conducted and used to propose cellular functions for individual cdG signaling genes. Finally, in-depth genetic analysis of an important rhizosphere colonization regulator revealed a link between cdG control of growth, motility and stress response, and the carbon sources available in the rhizosphere.

## Introduction

Members of the large *Pseudomonas* genus are colonizers of aquatic and terrestrial habitats, frequently associating with plants in a mutualistic, pathogenic, or saprophytic manner. Furthermore, all surfaces of the plant from root to tip and the soil environment influenced by plant exudates (the rhizosphere), are colonized in addition to endophytic occupation of interstitial spaces (Sitaraman, 2015). The rhizosphere and surface regions of a plant present a very different set of conditions compared to the surrounding bulk soil, and colonization of these environments demands a complex set of cellular responses. Plant colonization involves a series of distinct lifestyle choices, with chemotaxis and spatial colonization transitioning to biofilm formation, where cells abandon motility in favor of a sessile lifestyle (Walker et al., 2004). Bacteria colonizing the rhizosphere are also subject to a range of environmental challenges. For example, innate host immunity will be encountered, competition with other organisms will increase and colonization of above-ground surfaces may present a risk of desiccation (Beattie, 2011; Zamioudis and Pieterse, 2012; Lugtenberg et al., 2017). The benefit of surmounting these challenges is access to abundant nutrients and carbon sources provided by, for example, root exudates or the internal structure of the plant in the case of endophytes (Badri and Vivanco, 2009; Fatima and Senthil-Kumar, 2015).

Whilst *Pseudomonas* spp. can display opportunistic traits in relation to survival in disparate and changing environments, many are also specialist colonizers of particular hosts and niche habitats (Spiers et al., 2000). This adaptation exerts an evolutionary force that is reflected in a flexible accessory complement of genes that can comprise as much as 18% of the total individual genome (Silby et al., 2009, 2011; Ozer et al., 2014). Nevertheless, many aspects of plant association are common to all pseudomonads including control of motility and secretion systems, metabolic adaptation, nutrient uptake and biofilm formation through exopolysaccharide production. Thus, many genes underpinning these traits are component parts of the *Pseudomonas* core genome (Loper et al., 2012).

An immutable requirement of niche colonization is for adaptive outputs to be mutually coordinated, responsive to environmental cues and reversible. Whilst external signals are manifold and largely uncharacterized, cyclic-di-GMP (cdG) is a master second messenger in *Pseudomonas* spp. that is made and degraded according to the nature of the cellular surroundings defined by these cues leading to appropriate and timely integrated responses (Hengge, 2009). CdG is a circular RNA molecule produced from two GTP molecules by diguanylate cyclase (DGC) enzymes possessing a conserved GGDEF domain. Phosphodiesterase (PDE) enzymes containing a conserved EAL triad or HD-GYP domain hydrolyze a phosphodiester bond of cdG to produce a linear molecule termed pGpG or two GMP molecules, respectively (Hengge, 2009; Romling et al., 2013). DGC enzymes for the synthesis of cdG possess a conserved GGD/EEF motif at the active site, with the catalytically active GGEEF motif commonly found in *Pseudomonas* (Kulesakara et al., 2006). More unusually, an active DGC with an AGDEF motif has been reported *in Vibrio cholerae* and an active SGDEF variant in *Pectobacterium atrosepticum* (Perez-Mendoza et al., 2011; Hunter et al., 2014). PDE enzymes of the “EAL” class require, in addition to the EAL triad, 8 absolutely conserved residues within the domain required to coordinate two metal centers, a catalytic water molecule and oxygen atoms of the cdG phosphate (Tchigvintsev et al., 2010).

Most bacterial genomes contain multiple DGC and PDE genes with varied sensory and regulatory input domains; *Pseudomonas* species typically encode several dozen (Galperin, 2005). The emerging dogma postulates that cellular levels of cdG are maintained as localized pools that effect colocalized effector-target hubs, which may themselves interact with other GGDEF/EAL modules (Sarenko et al., 2017). Considering the large number of PDE and DGC genes in a typical *Pseudomonas* genome and the complexity of the environment being sensed, it is predictable therefore that genes encoding GGDEF and EAL proteins will not be transcribed en masse but rather in a manner that reflects the need for the specific output that they control at key stages of niche adaptation.

CdG acts at all levels of cellular activity to regulate transcription, mRNA translation, ribosome behavior and protein activity (Hengge, 2009; Romling et al., 2013; Grenga et al., 2017). In general terms, low intracellular concentrations of cdG are associated with flagellar-based motility and virulence including control of type III secretion. Conversely, elevated levels of cdG inhibit these processes and promote the biosynthesis of adhesins and exopolysaccharide matrix substances. During the colonization of the rhizosphere, it is to be expected that these processes will be variously required and not necessarily mutually exclusive. Such a model would further argue for cdG to act as temporally regulated pools that would in turn require dynamic regulation of genes for cdG synthesis and degradation throughout the colonization process. The model mutualistic plant colonizer *Pseudomonas fluorescens* SBW25 (henceforth called SBW25) possesses 45 predicted genes for cdG metabolism consisting of 25 DGC (GGDEF) genes, six PDE (five EAL, one HD-GYP) genes and 14 hybrid genes containing domain signatures for both synthesis and catabolism of the messenger (Silby et al., 2009).

We have identified eight cdG metabolism genes among a larger set of 147 SBW25 loci that are upregulated in the plant rhizosphere (Silby et al., 2009), and have characterized in detail the key role played by one of these operons in environmental adaptation and plant colonization through remodeling of the cellular proteome (Little et al., 2016). In this study we take a holistic approach to determine the contribution of the seven remaining genes throughout the plant colonization process. A detailed transcriptional and phenotypic analysis has been undertaken to illuminate the contribution that these seven cdG enzymes make to cellular adaptation. PFLU6074, a membrane-bound hybrid protein containing tandem PAS sensor domains, was shown to be particularly important for competitive rhizosphere colonization. A focused examination of this protein revealed a surprising link between cdG regulation of adaptive phenotypes and the available carbon source, implicating cdG in sensing and responding to the primary metabolic state of the bacterial cell. Mutants lacking this regulator also displayed markedly increased sensitivity to osmotic pressure, a phenotype that we ascribe to reduced osmoprotectant levels stemming from altered primary carbon metabolism.

## Materials and Methods

### Strains and Growth Conditions

Strains and plasmids are listed in Supplementary Table S1. Primers are listed in Supplementary Table S2. Unless otherwise stated all *P. fluorescens* strains were grown at 28°C and *E. coli* at 37°C in lysogenic broth (LB) (Miller, 1972), solidified with 1.5% agar where appropriate. Tetracycline (Tet) was used at 12.5 μg/ml, piperacillin and fosfomycin at 2 mg/ml and Congo Red at 0.004% w/v. For inducible plasmids, IPTG was added to a final concentration 1 mM as appropriate. 5-bromo-4-chloro-3-indolyl-β-D-galactopyranoside (XGal) was added to a final concentration of 40 μg/ml where appropriate.

### Molecular Biology Procedures

Cloning was carried out in accordance with standard molecular biology techniques. Overexpression plasmids were constructed by ligation of the appropriate gene PCR fragment (amplified from genomic DNA) between the *Eco*RI and *Kpn*I sites of plasmid pME6032 (Heeb et al., 2000). *P. fluorescens* SBW25 deletion mutants were prepared according to the protocol described previously (Little et al., 2016). Briefly, up- and downstream flanking regions to the target genes were amplified (using primers 15–46) and ligated into pME3087 between *Eco*RI-*Bam*HI. The resulting vectors were transformed into the target strain and single crossovers selected on Tet and re-streaked, before overnight growth in LB medium. Cultures were then diluted 1:100 into fresh medium and grown for 2 h before addition of 5 μg/ml Tet to stop the growth of double crossovers, then 2 mg/ml piperacillin and fosfomycin after a further hour to kill growing bacteria. Cultures were grown for a further 4–6 h, washed once in LB and a dilution series plated onto LB agar. Individual colonies were patched onto LB plates ± Tet, and gene deletion/modification tested for Tet-sensitive colonies by colony PCR.

### Reverse Transcriptase Quantitative PCR (RT-qPCR)

cDNA synthesis was performed using SuperScript II reverse transcriptase and random primers (Invitrogen) in the presence of RNasin ribonuclease inhibitor (Promega). Hundred nanogram of total RNA was used. cDNA was then used as template in qRT-PCR performed with a SensiFAST SYBR No-ROX kit (Bioline). Three liquid culture biological replicates and three rhizosphere biological replicates were used for each gene. Specific qPCR primers (numbers 1–14) were used to amplify reference and target genes. To normalize for differing primer efficiency, a standard curve was constructed (in duplicate) using chromosomal DNA. Melting curve analysis was used to confirm the production of a specific single product from each primer pair. qRT-PCR was performed using a CFX96 Touch instrument using hard-shell white PCR plates (Bio Rad). PCR products were detected with SYBR green fluorescent dye and amplified according to the following protocol: 95°C, 3 min, then 50 cycles at 95°C 5 s, 62°C 10 s and 72°C 7 s. Melting curves were generated at 65–95°C with 0.5°C increments. Primers were used at a final concentration of 250 nM.

### Rhizosphere Colonization

Paragon wheat seeds were sterilized with 70% ethanol and 5% hypochlorite, washed with distilled water and germinated on sterile 0.8% MS agar for 72 h in the dark. Seedlings were then transferred into sterile 50 ml tubes containing medium grain vermiculite and rooting solution (1 mM CaCl_2_⋅2H_2_O, 100 μM KCl, 800 μM MgSO_4_, 10 μM FeEDTA, 35 μM H_3_BO_3_, 9 μM MnCl_2_⋅4H_2_O, 0.8 μM ZnCl_2_, 0.5 μM Na_2_MoO_4_⋅2H_2_O, 0.3 μM CuSO_4_⋅5H_2_O, 6 mM KNO_3_, 18.4 mM KH_2_PO_4_, and 20 mM Na_2_HPO_4_), and transferred to a controlled environment room (25°C, 16 h light cycle). WT-*lacZ* and mutant SBW25 strains were grown overnight in M9 0.4% pyruvate media, then diluted in phosphate buffer and 1 × 10^3^ CFU of mutant and WT-*lacZ* bacteria used to inoculate 7 days old seedlings. Plants were grown for a further 7 days, after which shoots were removed, 20 ml PBS was added to each tube and vortexed thoroughly to resuspend bacteria. A dilution series was plated onto XGal + IPTG plates and WT-*lacZ*/mutant colonies distinguished by blue/white selection. Assays were conducted for 8–12 plants/mutant, repeated at least twice independently, and statistical significance assessed using Mann–Whitney *U*-tests.

### Wheat Root Attachment

SBW25 strains containing the bioluminescent plasmid pIJ-11-282 (Pini et al., 2017) were grown overnight at 28°C in M9 0.4% pyruvate media. Cultures were normalized for luminescence using a GloMax Multi JR luminometer (Promega) and diluted 1:100 in 10 ml 25 mM pH7.5 phosphate buffer in sterile 50 ml tubes, each containing 12–15 1.5 cm long sterile 3 days old wheat root tips (germinated as for rhizosphere colonization, above). Tubes were incubated for 2 h at room temperature with gentle shaking, before supernatant was discarded and the roots washed twice with phosphate buffer. 10 roots per sample were transferred to individual tubes and luminescence measured and compared with that obtained for wild-type SBW25. The assay was repeated twice independently, and statistical significance assessed using Student’s *t*-tests.

### Growth Assays

Bacterial growth was monitored in a microplate spectrophotometer (BMG Labtech/Biotek) using a minimum of 4 experimental replicates/sample. Wells (of a 96-well plate) contained 150μL of the indicated growth medium, supplemented with IPTG and tetracycline where appropriate. Growth was initiated by the addition of 5μL of cell culture with an OD_600_ = 0.01. Plates were either incubated statically at 28°C or under continuous agitation (Supplementary Figures S1, S2) comprising 200 rpm shaking rising to 500 rpm for 5 min prior to hourly data acquisition. Absorbance was measured at 600 nm; fluorescence, where relevant was monitored at 460 nm with an excitation wavelength of 355 nm. The carbon source indicated on the figure was present at a concentration of 0.4% w/v in each case. Casamino acids (Duchefa Biochemie) were present at 0.4% w/v where indicated. Assays were repeated at least once independently.

### Swarming Morphology and Swarm Diameter Assays

Kings B (KB) media plates (King et al., 1954) were prepared containing 0.4% w/v agar, 1% w/v NaCl, 0.004% Congo Red dye, with tetracycline added where relevant to maintain overexpression plasmids. Plates were allowed to set and dry for 45 min in a sterile flow chamber. SBW25 strains were grown overnight in LB media (with Tet as necessary) to stationary-phase. Cell densities were normalized then 1.5 μL of each culture was spotted onto the media surface. Triplicate plates were prepared. Plates were then incubated overnight at 28^o^C before photographing. To quantify swarming diameter for SBW25 cdG-gene mutants, 0.3% M9- agar plates were prepared containing the carbon sources indicated at a concentration of 0.4% w/v. 1.5 μL of early stationary-phase cultures was spotted onto the media surface and swarm diameter measured after 16 h of incubation.

### Random Transposon Mutagenesis

SBW25 Δ*PFLU6074*Δ*rccA* was transformed using the mariner plasmid pALMAR3 as described previously (Malone et al., 2010). Mariner transformants were initially selected on LB supplemented with tetracycline. Transposon insertions resulting in the recovery of isogenic swarming behavior were screened primarily on KB, and secondarily using M9 swarming plates with pyruvate as a carbon source. The location of the transposon insertion in each case was determined by arbitrary PCR (O’Toole and Kolter, 1998) using the following primer pairs: Arb-PCR and Arb1 and Arb1b and Almar3-seq (Supplementary Table S2).

## Results

### Eight cdG Metabolic Genes Are Associated With Sugar Beet Upregulated Loci in SBW25

**FIGURE 1:**
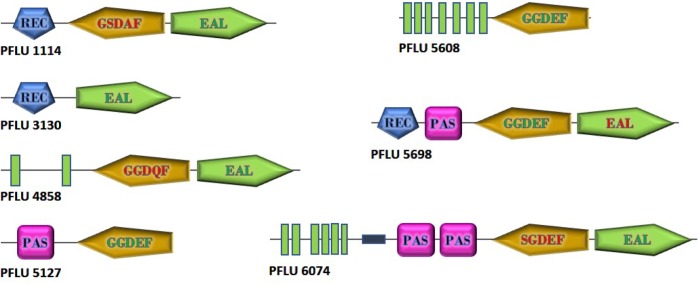
Domain structure of cdG metabolizing genes from *Pseudomonas fluorescens* SBW25 upregulated in the plant rhizosphere. GGDEF and EAL domains predicted to be active are shown with green lettering. Degenerate GGDEF domains are shown with red lettering and the non-consensus sequence is displayed. EAL domains lacking the eight additional conserved residues required for catalytic function are shown in red. Green boxes represent transmembrane regions. The relative size of the proteins is as shown. Domain architectures are based on SMART (https://smart.embl.de/) representations.

To investigate the role of cdG metabolism in plant rhizosphere colonization, we screened our existing *In Vivo* Expression Technology (IVET) dataset of sugar beet upregulated SBW25 loci for cdG metabolic genes (Silby et al., 2009). Eight cdG-associated genes were identified as either directly upregulated (*PFLU0263*, *PFLU4858*, *PFLU5608*), part of an upregulated operon (*PFLU1114*, *PFLU5698*) or immediately adjacent to an upregulated locus (*PFLU6074*, *PFLU3130*, *PFLU5127*). One of these genes, *PFLU0263* (*rimA*) has already been studied extensively and shown to play an important role in rhizosphere adaptation (Little et al., 2016). Bioinformatic analysis revealed that three of the remaining genes contain DGC domains with a conserved GGDEF motif (*PFLU5127*, *PFLU5608*, *PFLU5698*) and four genes contain PDE domains with the EAL triad and eight additional conserved residues (*PFLU1114*, *PFLU3130*, *PFLU4858*, *PFLU6074*) (Figure 1). In the case of the hybrid proteins *PFLU1114*, *PFLU4858*, and *PFLU5698* the alternative domain is degenerate and likely to provide a regulatory function, such as protein interaction or possibly cellular localization (Hengge, 2009). Finally, *PFLU6074* encodes a conserved EAL domain and a GGDEF domain with an SGDEF motif, which is hypothetically enzymatically competent (Perez-Mendoza et al., 2011). The gene product of *PFLU4858* possesses 82% identity with BifA, a previously characterized hybrid enzyme with phosphodiesterase activity (Kuchma et al., 2007). Additionally, *PFLU5698* possesses 67% identity and identical domain organization with the root-upregulated diguanylate cyclase Rup4959 from *Pseudomonas putida* (Matilla et al., 2011; Ramos-Gonzalez et al., 2016). Hereafter, *PFLU4858* will be referred to as *bifA*.

### Plant-Upregulated cdG Genes Are Differentially Transcribed Throughout the Colonization Process and Control Phenotypes Associated With Rhizosphere Adaptation

We have previously shown that expression of the *rim* locus (containing *PFLU0263*) is upregulated during the initial stages of wheat rhizosphere colonization (Little et al., 2016). In order to confirm that the remaining seven genes are upregulated in the plant environment and to determine their transcriptional profiles during colonization, SBW25 cells were recovered following either 1- or 7 days exposure to the root environment of wheat seedlings. RNA was recovered, and RT-qPCR performed to probe mRNA abundance for the genes of interest.

**FIGURE 2:**
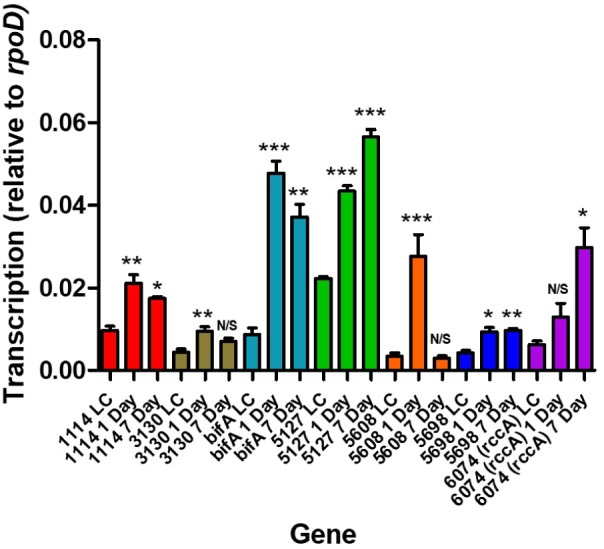
RT-qPCR analysis of rhizosphere-upregulated genes showing transcript abundance during 1- and 7 days colonization of model wheat rhizospheres relative to transcription in liquid culture (denoted LC). Statistically significant differences between transcript levels during colonization and the respective liquid culture control were analyzed using Dunnett’s Multiple Comparison ANOVA test with a minimal requirement of *P* < 0.05 (^∗^*P* ≤ 0.05; ^∗∗^*P* ≤ 0.01; ^∗∗∗^*P* ≤ 0.001). N/S, not significant.

Relative to growth in defined liquid culture containing pyruvate as a carbon source, all seven genes revealed an upregulation as a consequence of occupying the rhizosphere environment (Figure 2). Interestingly, three distinct patterns of regulation were seen; namely, transcription continued to increase over 7 days (in the case of *PFLU5127* and *PFLU6074*), transcription rose and fell over 7 days (in the case of *PFLU1114*, *PFLU3130*, *bifA* and most dramatically in the case of *PFLU5608*) or transcription rose and was maintained over 7 days (in the case of *PFLU5698*). This clearly suggests that the activities of specific enzymes for cdG synthesis or catabolism are required at different stages of rhizosphere colonization.

**FIGURE 3:**
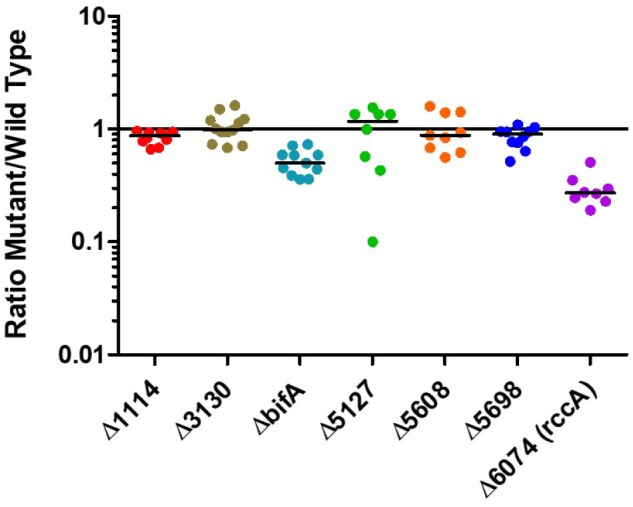
Rhizosphere colonization competition assays. The graph shows the ratio of mutant strains lacking a cdG metabolizing gene to wild-type-*lacZ* SBW25 colony forming units recovered from model wheat rhizospheres 7 days post-inoculation.

Having established that the selected genes were indeed responsive to the plant environment we sought to determine how strains of SBW25 lacking these genes would compete with the wild type strain in model wheat rhizospheres (Figure 3). Strikingly, mutant *PFLU6074* (a putative phosphodiesterase) was much less competitive than wild type cells as was a mutant in *bifA*, another putative phosphodiesterase. Deletion of the other genes did not produce significant differences in CFU abundance after 7 days.

**FIGURE 4:**
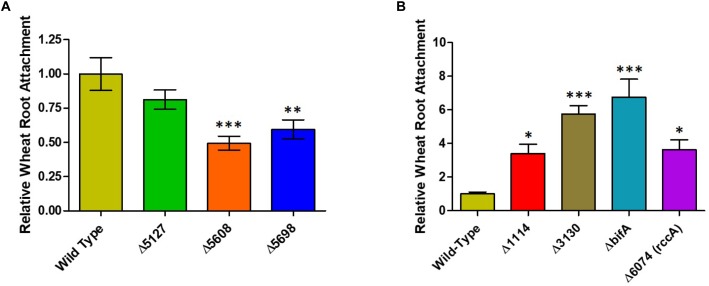
**(A)** Wheat root attachment assay. The ability of SBW25 cells lacking a putative DGC gene to adhere to wheat roots is plotted relative to wild-type cells. **(B)** The ability of SBW25 cells lacking a putative PDE gene to adhere to wheat roots is plotted relative to wild-type cells. Statistically significant differences between transcript levels during colonization and the respective liquid culture control were analyzed using Dunnett’s Multiple Comparison ANOVA test with a minimal requirement of *p* < 0.05 (^∗^*P* ≤ 0.05; ^∗∗^*P* ≤ 0.01; ^∗∗∗^*P* ≤ 0.001). Columns without an asterisk do not differ significantly from the control.

Whilst our competition assay gives some indication of the relative ability of SBW25 mutant strains to replicate in the rhizosphere, it does not provide insights into the specific aspects of plant colonization that may be affected by the mutation. To address this question, we first conducted root attachment assays with the mutant strains to determine their ability to adhere to the biotic surface. Strikingly, deletion of genes for all three putative DGC enzymes (*PFLU5127*, *PFLU5608*, and *PFLU5698*) resulted in reduced ability to adhere to the root surface (Figure 4A) whereas deletion of the putative PDE enzymes revealed an enhanced ability to surface-attach relative to wild type cells (Figure 4B). The *bifA* mutant displayed the greatest degree of root attachment consistent with the hyper-adherent phenotype demonstrated for the equivalent mutation in *P. putida* (Jimenez-Fernandez et al., 2015). Thus, the attachment behaviors observed for our mutants are consistent with the assignment of each gene as encoding either an active DGC or PDE.

**FIGURE 5:**
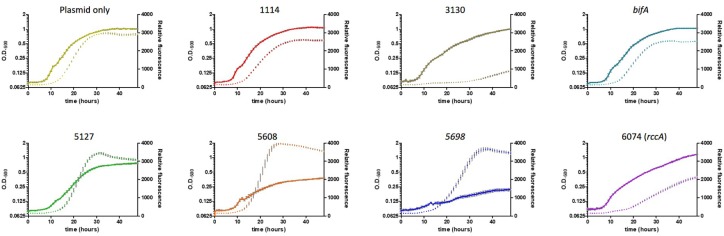
Growth of SBW25 cells overexpressing a gene for CGD metabolism with simultaneous acquisition of pyoverdine fluorescence in M9-pyruvate media. Solid line: optical density at 600 nm. Dashed line: relative fluorescence measured at 460 nm (excitation: 355 nm). Error bars show the standard error of the mean. The gene identity (*PFLU*) number or name of the gene that has been overexpressed is displayed above each panel.

As its name suggests, *Pseudomonas fluorescens* possesses a fluorescent property conferred by the siderophore pyoverdine. This soluble pigment chelates inorganic ferric ions and contributes to disease suppression and plant immunity by some strains of plant growth promoting rhizobacteria (Haas and Defago, 2005; Aznar and Dellagi, 2015). Pyoverdine synthesis has been shown to be positively regulated by DGC activity (Malone et al., 2010; Chen et al., 2015). To investigate the role of the cdG-metabolic genes on siderophore production, we conducted growth experiments *in vitro* whereby rate of growth and associated fluorescence were simultaneously measured, allowing us to determine if cell division or siderophore production, respectively, were affected by overexpression of the cdG metabolizing genes (Figure 5).

Cell growth in defined media containing pyruvate as carbon source was severely reduced upon overexpression of the putative DGC genes *PFLU5608* and *PFLU5698*, however, fluorescence (relative to cell density) was greatly enhanced for all three putative DGC genes (*PFLU5127*, *PFLU5608*, *PFLU5698*) relative to the vector-only control. Conversely, pyoverdine production was drastically reduced upon overexpression of *PFLU3130*, and lessened upon overexpression of *PFLU1114*, *bifA*, and *PFLU6074*, consistent with the designation of these genes as encoding active phosphodiesterase enzymes (Figure 5).

**FIGURE 6:**
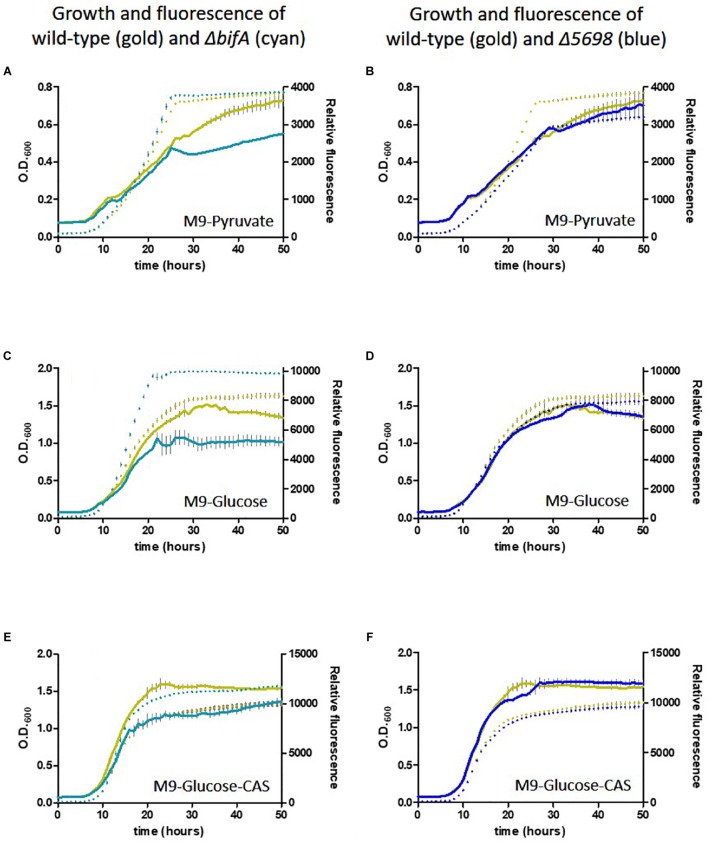
Growth of SBW25 deletion mutants with simultaneous acquisition of pyoverdine fluorescence in M9 media with the indicated carbon source or supplement. Solid line, optical density at 600 nm; dashed line, relative fluorescence measured at 460 nm (excitation: 355 nm). Error bars show the standard error of the mean. Wild-type SBW25 is shown in gold, Δ*bifA* is shown in cyan (left-hand panels), Δ*PFLU5698* is shown in dark blue (right-hand panels). **(A,B)** Growth in M9-Pyruvate media; **(C,D)** Growth in M9-Glucose media; **(E,F)** Growth in M9-Glucose-Casamino acids media.

We conducted a similar experiment using the single in-frame deletion strains for these genes in the presence of different carbon and nutrient sources. Deletion of five genes had little or no influence on growth or siderophore production. Notably, whilst growth was negatively affected, fluorescence was increased upon deletion of *bifA*, most strikingly in the presence of glucose as carbon source and glucose plus casamino acids. Deletion of *PFLU5698* resulted in notably reduced fluorescence in the M9-pyruvate condition (Figure 6). These results suggest that *bifA* and *PFLU5698* perform major roles in modulating siderophore production.

To investigate the possibility that these relative behaviors may be dependent upon oxygen concentration, the experiments were repeated with sustained agitation to maintain a constant level of aeration. Cells overexpressing the genes of interest entered stationary phase sooner and therefore achieved a lower optical density (Supplementary Figure S1). Production of the fluorescent siderophore, pyoverdine was apparently promoted under these conditions. However, the pattern of behavior relative to the plasmid-only control, was essentially maintained albeit less pronounced.

Sustained agitation of the deletion mutants Δ*bifA* and Δ*PFLU5698* produced the same pattern of behavior relative to wild-type cells although once again the differences were less pronounced (Supplementary Figure S2).

**FIGURE 7:**
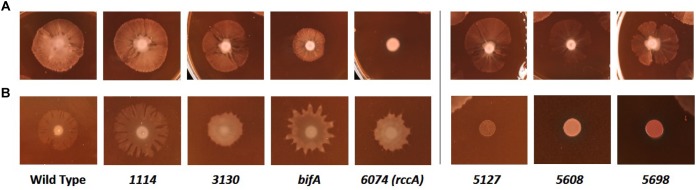
Kings B-Congo Red morphology plates. **(A)** SBW25 deletion mutants are compared to wild-type cells. **(B)** SBW25 cells overexpressing cdG metabolizing genes are compared to wild-type cells containing an “empty” overexpression plasmid. The deleted **(A)** or overexpressed **(B)** gene is denoted below the relevant panels.

We next sought to determine the influence of the cdG genes on colony morphology and swarming behavior on semi-solid media. Deletion of five genes had little or no impact on the morphology or extent of swarming motility under our plate conditions (Figure 7A). However, deletion of *bifA* resulted in a marked reduction in bacterial swarming, consistent with results seen for the *P. aeruginosa* homolog (Kuchma et al., 2007), while deletion of *PFLU6074* resulted in an almost complete loss of swarming under our test conditions (Figure 7A). Overexpression of *PFLU5127, PFLU5608*, and *rup4979* strongly inhibited swarming resulting in an auto-aggregative small colony variant morphotype consistent with DGC overexpression and in line with results seen for the *P. putida* PFLU5698 homolog (Matilla et al., 2011; Malone, 2015). Furthermore, *PFLU5698* overexpression strongly increased Congo Red binding indicating elevated cdG-regulated synthesis of exopolysaccharide (Figure 7B). Overexpression of *PFLU3130, bifA*, and *PFLU6074* resulted in unusual swarming phenotypes, again suggesting roles for these proteins in the control of motility (Figure 7B).

### The Δ*PFLU6074* Mutant Phenotype Can Be Recovered by Disrupting the Regulation of Primary Carbon Metabolism

At this stage we decided to examine the *PFLU6074* gene product in more detail, based on its marked impacts on competitive rhizosphere colonization and swarming motility. *PFLU6074* is contiguous with the *rccR* gene (*PFLU6073*), which encodes a transcriptional regulator of primary carbon metabolism that was previously characterized by this laboratory (Campilongo et al., 2017), and which itself plays an important role in competitive rhizosphere colonization. RccR controls flux through the glyoxylate shunt pathway to allow growth on simple carbon compounds, in response to levels of the Entner Douderoff pathway intermediate 2-dehydro-3-deoxy-phosphogluconate (KDPG). The metabolic flexibility permitted by RccR is a critical enabler of adaptation to the rhizosphere that presents a mixture of carbon sources including simple sugars and organic acids derived from root exudate (Walker et al., 2003).

**FIGURE 8:**
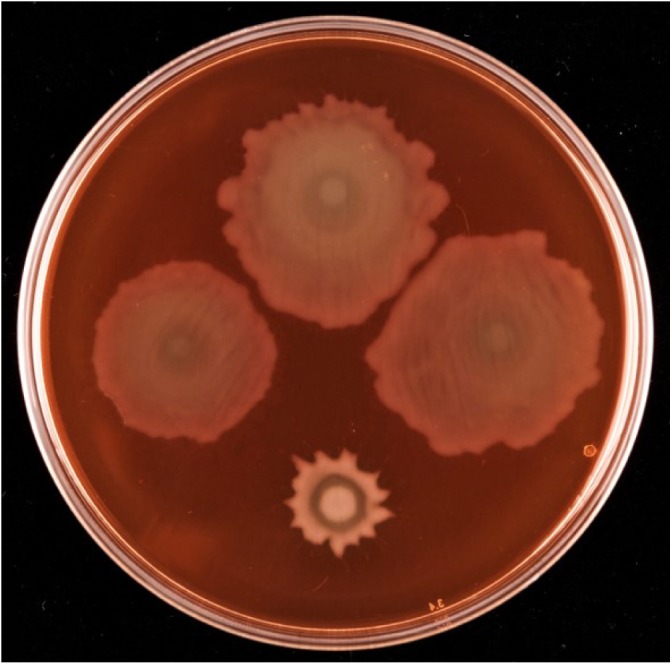
Swarming motility of SBW25 single and double *rcc*-deletion strains on M9 media (lacking NaCl in this instance), compared to wild-type cells. Clockwise from top, wild-type SBW25, Δ*rccR*, Δ*rccA*, Δ*rccR*/Δ*rccA*.

Given the proximity on the chromosome of *PFLU6074* (henceforth named *rccA*) to *rccR*, their similar effects on rhizosphere colonization and the two tandem PAS domains of RccA that suggest a role in environmental sensing (Figure 1), we reasoned that regulation by the two proteins may overlap in some way. To test this hypothesis, we produced a *rccAR* double mutant and compared its swarm phenotype to single deletion mutants of *rccA* and *rccR.* As shown previously, deletion of *rccA* produced a highly distinctive swarming phenotype on KB medium, whereas deletion of *rccR* produced swarm behavior that appeared very similar to that of wild-type cells (Figure 8). Strikingly, the Δ*rccA* phenotype could be substantially recovered by the additional deletion of *rccR* suggesting that the RccA and RccR regulons are indeed genetically linked.

**FIGURE 9:**
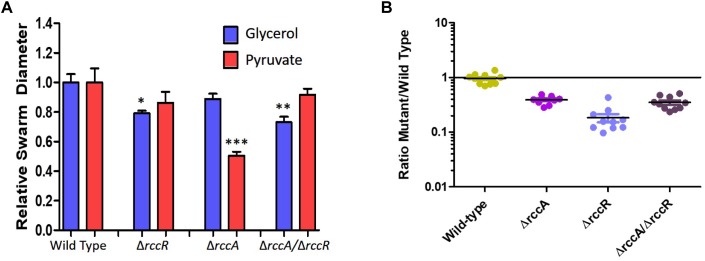
**(A)** Swarming motility of SBW25 single and double *rcc*-deletion strains compared to wild-type cells on M9 media in the presence of pyruvate or glycerol as carbon source. Statistically significant differences between swarm diameter relative to the appropriate wild-type control were analyzed using Dunnett’s Multiple Comparison ANOVA test with a minimal requirement of *p* < 0.05 (^∗^*P* ≤ 0.05; ^∗∗^*P* ≤ 0.01; ^∗∗∗^*P* ≤ 0.001). Columns without an asterisk do not differ significantly from the control. **(B)** Rhizosphere colonization competition assays. The graph shows the ratio of mutant strains lacking a cdG metabolizing gene to wild-type-*lacZ* SBW25 colony forming units recovered from model wheat rhizospheres 7 days post-inoculation. The wild-type control shows the ratio of wild-type cells to wild-type-*lacZ* cells confirming the neutrality of the *lacZ* marker.

As RccR is a regulator of primary carbon metabolism we sought to investigate the possibility that the distinctive *rccA* swarm behavior may be responsive to the available carbon source. Further motility assays were performed on defined carbon sources, and the resulting swarm diameters measured (Figure 9A). Deletion of *rccR* had little effect on swarm diameter in the presence of pyruvate and a small but significant reduction in glycerol. Deletion of *rccA*, however, resulted in drastically reduced swarming in the presence of pyruvate. Remarkably, this phenotype was wholly recovered by the additional deletion of *rccR*. In the presence of glycerol, the *rccAR* double mutant resulted in a modest reduction in swarm diameter, suggesting that the *rccR* deletion is dominant over Δ*rccA*. The recovery of a wild-type morphotype by the *rccAR* double mutant did not extend to rhizosphere colonization, with the *rccAR* strain performing, similarly, to the *rccA* mutant in competitive wheat colonization assays (Figure 9B).

To further investigate the link between RccA activity and primary carbon metabolism, we conducted a transposon mutagenesis screen for suppressor mutations of the Δ*rccA* swarming phenotype. A total of 3,511 transposon mutants of the Δ*rccA* strain were screened on KB and then M9 pyruvate swarm plates and those colonies that displayed a wild-type swarm pattern on both media were subjected to semi-random PCR sequencing to identify the position of insertion on the chromosome. In the majority of cases, colonies displaying wild-type swarm behavior carried insertions in genes involved in carbon metabolism, including acetyl-CoA synthetase involved in acetate utilization. Additionally, genes for nitrogen/carbon metabolism, the *kinB* histidine kinase (Barahona et al., 2011) and a GGDEF/EAL-encoding gene [*PFLU5329, morA* (Liu et al., 2018)] were also disrupted (Table 1). Intriguingly, two recovery mutants mapped to the same genomic region within the phage integrase gene *PFLU0179*. Disruption of this gene had no obvious role in suppression of the Δ*rccA* phenotype. However, analysis of the upstream region of the gene revealed the presence of a previously unidentified binding site for the RccR paralog HexR.

**Table 1:** Transposon-disrupted genes identified from SBW25 Δ*rccA* cells displaying a wild-type-like swarm motility.

Gene hit	Distance from 5′	Promoter direction	Gene function
*PFLU0179*	36 aa	Plus	Putative phage integrase
*PFLU0179*	N/D	N/D	Putative phage integrase
*PFLU0920*	130 aa	Plus	Glycerate dehydrogenase
*PFLU1255*	612 aa	Plus	Glycerol-3-phosphate acyltransferase
*PFLU1268*	749 aa	Minus	PII-uridylyl-transferase (*glnD*)
*PFLU1513*	N/D	Minus	Putative glutamine synthetase
*PFLU4766*	138 aa	Minus	Acetyl-CoA synthetase
*PFLU4766*	399 aa	Plus	Acetyl-CoA synthetase
*PFLU0087*	1 aa	Plus	Two-component system sensor kinase (*kinB*)
*PFLU5329*	183 aa	Minus	Putative sensory box GGDEF/EAL domain protein
*PFLU0156*	40 aa	Minus	Hypothetical protein
*PFLU0426*	354 aa	Minus	Conserved hypothetical protein


*N/D, not determined. Promoter direction refers to the direction of the Tet promoter in relation to the disrupted open reading frame. Gene function refers to annotated functions according to www.pseudomonas.com (Winsor et al., 2011).*

**FIGURE 10:**
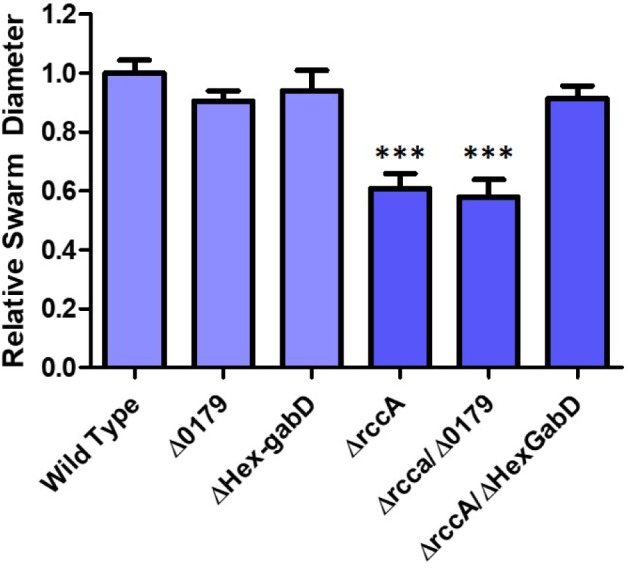
Swarming motility on M9 media, relative to wild-type cells, of SBW25 strains possessing deletion of *rccA*, *PFLU0179*, mutation of the *Hex-gabD* binding site or combinations of these backgrounds in the presence of pyruvate as carbon source. Statistically significant differences between swarm diameter relative to the appropriate wild-type control were analyzed using Dunnett’s Multiple Comparison ANOVA test with a minimal requirement of *p* < 0.05 ^∗∗∗^ denotes *P* ≤ 0.001Columns without an asterisk do not differ significantly from the control.

HexR is structurally almost identical to RccR and coordinately regulates glucose metabolism through repressive binding to a consensus DNA sequence (5′-TTGT-N_7-8_-ACAA-3′) mediated by the SIS (sugar isomerase) domain ligand KDPG (Campilongo et al., 2017). The orientation and location of the putative HexR binding site is consistent with regulation of gene *PFLU0180* (*gabD*) encoding succinate-semialdehyde dehydrogenase, which converts succinate-semialdehyde to succinic acid for entry into the Krebs cycle (Albers and Koval, 1961). We therefore postulated that recovery of the *rccA* swarming phenotype may arise through polar disruption of HexR repression, and consequent *gabD* transcription. To test this hypothesis, the putative HexR binding site was mutated to disrupt the consensus sequence (from 5′-TTGT-N_7_-ACAA-3′ to 5′-**C**TGT-N_7_-**G**CA**G**-3′) and swarming assays were performed on M9 pyruvate plates. Little or no effect was observed on the swarm diameters of strains either lacking *PFLU0179* or containing the disrupted HexR consensus sequence alone (Figure 10), and a Δ*rccA*/*PFLU0179* double mutant retained the *rccA* swarming phenotype. However, the Δ*rccA* swarming deficiency could be fully reversed by the HexR consensus mutation.

**FIGURE 11:**
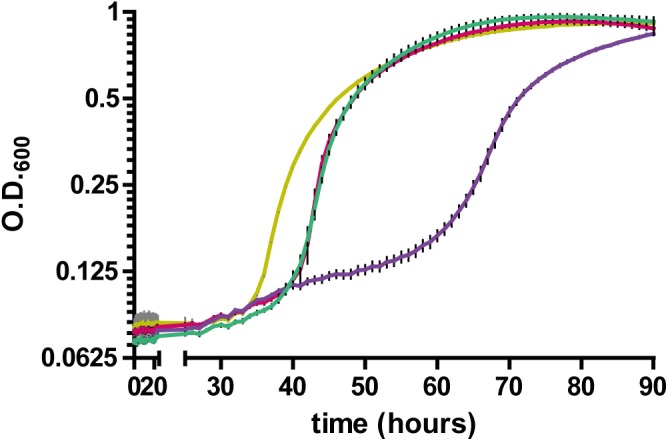
Growth of SBW25 Δ*rccA*, Δ*rccR* and double mutant strains relative to wild-type cells in M9-Pyruvate media, under conditions of osmotic challenge. NaCl was present at a concentration of 0.85M. Gold line, wild-type; red line, Δ*rccR*, green line, Δ*rccA*/ Δ*rccR;* purple line, Δ*rccA*. Error bars show the standard error of the mean.

Having established a link between RccA and metabolic regulation by RccR and HexR, we reasoned that cells lacking the Δ*rccA* mutant might display differential sensitivity to external stress as a consequence of altered carbon metabolism (Sevin et al., 2016). To test this hypothesis, we conducted growth assays in M9 media containing pyruvate and 0.85M sodium chloride. Δ*rccR* SBW25 cells exhibited a slightly extended lag period but otherwise grew, similarly, to wild-type cells (Figure 11). Strikingly however, the Δ*rccA* mutant was highly sensitive to the presence of salt; whilst the mutant was eventually able to reach a comparable density to wild-type, lag phase for this strain was markedly extended. Similar to the swarming phenotype data above (Figure 8), the Δ*rccAR* double mutant had an essentially identical pattern of growth to Δ*rccR* SBW25 (Figure 11).

We reasoned that if the metabolic outcome of mutating the HexR consensus sequence upstream of *gabD* is comparable to the influence of RccA activity on carbon metabolism then introduction of this mutation into the Δ*rccA* mutant should prevent a hypersensitive response to osmotic stress. To test this hypothesis, a further growth assay in M9 media containing pyruvate and 0.85M sodium chloride was performed. Whilst the Δ*Hex-gabD* strain revealed wild-type sensitivity to the presence of NaCl, the introduction of the Δ*Hex-gabD* mutation into the Δ*rccA* mutant significantly attenuated the osmotic sensitivity of Δ*rccA* strain (Supplementary Figure S3). Combining the Δ0179 mutation with Δ*rccA* had no ameliorating effect and actually increased sensitivity to NaCl.

## Discussion

In this study we have confirmed the assignment of seven cdG metabolism genes as upregulated in plant rhizospheres and assessed their importance for rhizosphere colonization and control of key bacterial phenotypes. Bioinformatic analysis allowed us to predict the probable enzymatic activity of the encoded proteins in terms of synthesis or degradation of cdG (Figure 1). Interestingly, all proteins are either located at the cytoplasmic membrane or possess sensory PAS or REC domains; in some cases, combinations of these. Thus, it is probable that all seven proteins are responsive either directly or indirectly, to environmental stimuli.

Determination of transcript abundance at 1- and 7 days into SBW25 colonization of model wheat rhizospheres revealed a complex, differential pattern of regulation for these genes. This result clearly demonstrates that both diguanylate cyclase (GGDEF) enzymes and phosphodiesterase (EAL) enzymes are required at each stage of rhizosphere adaptation (Figure 2). This is consistent both with the complex nature of the rhizosphere environment and with recent evidence that levels of cdG metabolizing proteins vary with growth phase and interact as discrete modules to determine specific phenotypic outputs (Sarenko et al., 2017).

Whilst the contribution of the genes to overall survivability *per se* in model rhizospheres was variable (Figure 3), deletion or overexpression of individual genes produced an output that was consistent with a role in modulating cellular cdG levels and with plant adaptation (Figure 5–7). Thus, deletion of all four putative EAL enzymes resulted in a greater capacity to adhere to root surfaces whereas deletion of the putative GGDEF proteins (in particular *PFLU5608* and *PFLU5698*) reduced the ability of cells to attach (Figure 4). This suggests that the two classes of enzyme make delineated and opposing contributions to plant colonization that reflect their respective abilities to modulate cdG concentrations. Attachment to a biotic surface is a multi-faceted process involving for example, reduction in motility, initial reversible attachment and subsequent irreversible attachment leading to biofilm formation. This requires the synthesis of many adherence factors including pili, adhesins and exopolysaccharides (Rodriguez-Navarro et al., 2007). Therefore, while we can unambiguously ascribe a cellular function to the putative phosphodiesterase and diguanylate cyclase enzymes in disfavoring or favoring root attachment, respectively, the exact process or processes under control of each enzyme remain the subject of future study.

Intriguingly, *PFLU5608* and *PFLU3130* are upregulated in the early stages of rhizosphere colonization, before transcript abundance drops to levels that are statistically indistinguishable from the liquid culture control by day 7; this behavior is particularly marked for *PFLU5608* (Figure 2). This would suggest that the activity of the encoded proteins is relevant to the early stages of colonization in our model rhizosphere assay. Deletion of *PFLU5608* most strongly reduces the ability of SBW25 to attach to root surfaces, while *PFLU3130* deletion led to a particularly strong attachment increase, suggesting a role for these proteins in particular in controlling initial root attachment (Figure 4A).

Biofilm development is driven by elevated intracellular levels of cdG and is also signaled by the presence of iron (Banin et al., 2005; Valentini and Filloux, 2016). Iron starvation can prevent bacterial growth and SBW25 synthesizes the high-affinity siderophore pyoverdine in order to scavenge ferric iron from the environment (Ringel and Bruser, 2018). Pyoverdine is not only important for sustaining cell viability but also serves as an indirect and direct virulence factor (Haas and Defago, 2005; Lugtenberg and Kamilova, 2009; Kang et al., 2018). The production of pyoverdine has further been shown to be positively regulated by cdG (Malone et al., 2010; Chen et al., 2015). Overproduction of the putative DGC enzymes (PFLU5127, 5608, and PFLU5698) resulted in a large increase in fluorescence relative to cell growth indicating increased synthesis of pyoverdine (Figure 5). Interestingly, cell growth was arrested in all three strains and severely so in the strains overexpressing *PFLU5608* and *PFLU5698*. In addition to controlling motility, biofilm formation, virulence and cell cycle progression, cdG can also inhibit cell division as a stress response that may be linked to iron-siderophore uptake (Barchinger and Ades, 2013; Kim and Harshey, 2016). Conversely, overproduction of the putative EAL enzymes (PFLU1114, PFLU3130, BifA, and RccA) led to a reduction in fluorescence relative to cell growth. While the rate of growth was also reduced in the latter three strains, perhaps as a non-specific response to protein overproduction, all strains reached the same final density as the wild-type control (Figure 5). Repetition of the growth experiments under conditions of continuous agitation to maintain a constant oxygen concentration suggested that whilst oxygen levels exerted an influence on cell growth and pyoverdine production *per se*, the gene products were not directly oxygen-sensitive.

This leads us to conclude that the relative response of the gene products will be essentially invariant with fluctuating oxygen concentrations in the rhizosphere. All seven cdG metabolizing genes possess the capacity to regulate pyoverdine synthesis. However, the extremes of cdG concentration resulting from overexpression of GGDEF or EAL proteins may well override local signaling circuits, meaning that control of pyoverdine synthesis cannot be ascribed to a specific module of GGDEF and EAL enzymes using this approach alone. Interestingly, deletion analysis distinguished the putative PDE BifA and the putative DGC PFLU5698 in modulating cellular fluorescence, suggesting that the activity of these two enzymes in particular controls pyoverdine production during rhizosphere colonization (Figure 6). The resulting modulation of cellular iron levels may be connected to the biofilm formation and root attachment phenotypes observed for the *bifA* and *PFLU5698* deletion mutants (Figure 4A,B; Banin et al., 2005). Interestingly, both *bifA* and *PFLU5698* have similar transcriptional profiles, maintaining mRNA synthesis throughout the 4 days monitoring period of rhizosphere colonization thus increasing the likelihood that the gene products work in a complimentary manner (Figure 2). Nevertheless, BifA activity appears to be more decisive in terms of generating a phenotypic output, reflected in the relatively high level of transcription and its stronger effects on rhizosphere colonization and root attachment relative to PFLU5698 (Figure 2, 3, 4A,B).

Overexpression of six out of seven genes affected swarming motility in a manner consistent with their predicted roles as DGCs or PDEs. One gene (*PFLU1114*) produced no effect on swarming either upon deletion or overexpression, effectively excluding it from the motility control pathway. Similar to the results seen for pyoverdine production, two genes; *bifA* and *rccA* produced noticeable swarming defects upon gene deletion, implicating these proteins in motility control during rhizosphere colonization. Interestingly, *bifA* deletion affected all the phenotypes we examined in this study, highlighting its central role in controlling *Pseudomonas* behavior during host-microbe interaction.

Having competed to survive in the complex and dynamic physical environment of the rhizosphere, *Pseudomonas* spp. must adapt their metabolic repertoire to utilize the diverse and variable carbon sources provided by plants (Ramachandran et al., 2011; Chavarria et al., 2012). We have previously described a key transcriptional regulator, RccR that controls gene expression in response to carbon source availability and is essential for competitive survival in plant rhizospheres (Campilongo et al., 2017). We were intrigued to note that *rccA* encoding a putative phosphodiesterase protein was located immediately upstream of the *rccR* gene. Here we demonstrate that RccA also plays an important function in maintaining cell competitiveness in model rhizospheres.

Phenotypical and bioinformatic analysis suggests that RccA possesses an active phosphodiesterase domain and an unusual (possibly inactive), SGDEF-motif DGC domain. The role of cdG degradation in signaling by the putative phosphodiesterase, RccA is currently unclear. Nevertheless, it is reasonable to postulate that degradation of cdG by RccA is an output controlled by the N-terminal sensory regions of the protein. In addition to DGC activity, non-enzymatically active DGC domains fulfill a range of regulatory and allosteric functions based on RNA and nucleotide binding, and/or protein-protein interaction (Jenal et al., 2017). RccA also contains several membrane-spanning helices and two cytoplasmic sensory PAS domains upstream of the cdG-responsive regions (Figure 1). The PAS domain is a ubiquitous signaling module commonly found in cdG metabolizing proteins and demonstrated to bind a diverse range of effectors including C4 and C6 carboxylic acids (Henry and Crosson, 2011). Thus, the control of RccA function is likely to be complex and potentially subject to regulation by multiple signals.

Deletion of *rccA* results in a reduced swarming phenotype that is recovered by additional deletion of *rccR*. Two opposing arguments could provide a rationale for the carbon source-dependent growth phenotypes observed for the Δ*rccA* mutant that are explicitly linked to RccR activity. Firstly, RccA could act upstream of RccR to control *rccR* transcriptional activity according to the metabolic state of the cell. Alternatively, RccA could act downstream of RccR to respond to environmental cue(s) resulting from RccR activity. RccR transcriptional repression is controlled by the ED-intermediate KDPG, a precursor to pyruvate resulting from glucose metabolism in the ED pathway (Entner and Doudoroff, 1952). In the presence of pyruvate as sole carbon source, cellular levels of KDPG will be low leading to RccR repression of genes for pyruvate metabolism, de-repression of the glyoxylate shunt pathway and (presumably) low levels of Krebs Cycle intermediates. Deletion of *rccR* permits pyruvate metabolism, albeit in an inefficient manner due to the ongoing presence of glyoxylate anabolism (Campilongo et al., 2017). Our results suggest that the redistribution of primary carbon metabolic pathways resulting from *rccR* deletion negates the need for (or represses the activity of) RccA.

Our transposon screen for suppressors of the *rccA* swarming phenotype produced two main classes of mutants. Firstly, two mutants were isolated in genes that have previously been linked to control of motility in *Pseudomonas* species: *morA* and *kinB* (Barahona et al., 2011; Liu et al., 2018). We expected to isolate mutations of this type, where loss of one signaling protein is suppressed by loss of an antagonist working in the same pathway. Less predictably, but consistent with the Δ*rccAR* phenotype we also saw swarming recovery upon disruption of several other primary metabolic genes (Table 1). While the metabolic disruptions that lead from these mutations are difficult to confidently predict, it is tempting to speculate that the effects of each of these mutations may produce similar disruptions to the intracellular pool of Krebs cycle intermediates as seen upon *rccR* deletion.

Finally, disruption of the putative HexR binding site upstream of *gabD* restored Δ*rccA* mutant swarming to wild-type levels (Figure 10) and significantly heightened the sensitivity of the Δ*rccA* mutant to osmotic stress. Low levels of KDPG resulting from growth on pyruvate favor the repressive state of the HexR protein (Kim et al., 2008). Hence, disruption of the HexR binding site will relieve transcriptional inhibition of *gabD* resulting in increased production of the succinate semialdehyde dehydrogenase enzyme. The predicted increase in cellular succinate concentrations apparently influences the flow of carbon intermediates through the ED and glyoxylate pathways to compensate for the loss of *rccA* in the same manner as the *rccAR* double mutant. Taken together, our results support the hypothesis that RccA senses succinate or another downstream intermediate of the Krebs Cycle and is responsible for integrating cellular metabolic state with motility and root attachment during plant colonization. This protein and its function in the rhizosphere are the subject of ongoing investigation.

*Pseudomonas* spp. differentially regulate a range of genes for osmoadaptation resulting in the intracellular accumulation of various protective compounds including the primary osmolyte trehalose (Aspedon et al., 2006). The reduced ability of SBW25 Δ*rccA* grown on pyruvate only to tolerate osmotic shock conditions may feasibly reflect an inability to perform gluconeogenesis, a pathway controlled by RccR. Alternatively, less direct factors may be at play including altered gene regulation for biosynthesis of osmoprotectants as a consequence of differing metabolite composition. Nevertheless, intermediates of primary carbon metabolism including pyruvate and glyoxylate positively correlate with osmotolerance, highlighting the importance of tightly regulated metabolism to meeting external challenges.

In summary, our analysis has underscored the importance of orchestrated expression of numerous cdG metabolizing genes throughout the course of rhizosphere colonization to control diverse phenotypes including motility, root attachment and siderophore production. Furthermore, in addition to controlling processes that facilitate ultimate attachment and competitive colonization of root surfaces we have uncovered an exciting new role for cdG metabolism in *P. fluorescens*, in carbon-dependent regulation of growth, motility and stress response.

## Data Availability

All datasets generated for this study are included in the manuscript and/or the Supplementary Files.

## Author Contributions

RL, SW, and JM conceived and designed the study and wrote the manuscript. RL, SW, RC, SP, and JM analyzed the data. All authors conducted the experimental work.

## Conflict of Interest Statement

The authors declare that the research was conducted in the absence of any commercial or financial relationships that could be construed as a potential conflict of interest.
