# Supplementary material for: Differential Regulation of Genes for Cyclic-di-GMP Metabolism Orchestrates Adaptive Changes During Rhizosphere Colonization by Pseudomonas fluorescens
Source: Front Microbiol. 2019 May 16;10:1089. doi: 10.3389/fmicb.2019.01089 (PMC6531821; doi:10.3389/fmicb.2019.01089)
Supplement: Supplementary file 1 [file Table_1.DOCX]

| Table S1. Strains and plasmids used in this study. | | |
| --- | --- | --- |
|  | | |
| *Strains* | *Description* | *Reference* |
| *Pseudomonas* |  |  |
| SBW25 | Environmental *P. fluorescens* isolate | (Rainey and Bailey, 1996) |
| SBW25 *Δ1114* | SBW25 with *PFLU_1114* deleted | This study |
| SBW25 *Δ3130* | SBW25 with *PFLU_3130* deleted | This study |
| SBW25 *ΔbifA* | SBW25 with *PFLU_4858* deleted | This study |
| SBW25 *Δ5127* | SBW25 with *PFLU_5127* deleted | This study |
| SBW25 *Δ5608* | SBW25 with *PFLU_5608* deleted | This study |
| SBW25 *Δrup4959* | SBW25 with *PFLU_5698* deleted | This study |
| SBW25 *ΔrccA* | SBW25 with *PFLU_6074* deleted | This study |
| SBW25 *ΔrccR* | SBW25 with *PFLU_6073* deleted | (Campilongo et al., 2017) |
| SBW25 *ΔrccA/ΔrccR* | SBW25 with *PFLU_6074/6073* deleted | This study |
| SBW25 *Δ0179* | SBW25 with *PFLU_0179* deleted | This study |
| SBW25 *ΔHex-gabD* | SBW25 with mutated HexR binding site | This study |
| SBW25 *ΔrccA/0179* | SBW25 with *PFLU_6074/0179* deleted | This study |
| SBW25 *ΔrccA/ΔHex-gabD* | SBW25 with *PFLU_6074/*HexR site deleted | This study |
| *E. coli* |  |  |
| BL21(DE3)pLysS | F^–^ *omp*T *hsd*S_B_ (r_B_^–^, m_B_^–^) *gal dcm* (DE3) pLysS(Cam^R^) | Novagen |
| DH5α | F^–^ φ80*lac*ZΔM15 Δ(*lac*ZYA-*arg*F)U169 *rec*A1 *end*A1 *hsd*R17(r_K_^–^, m_K_^+^) *pho*A *sup*E44 λ^–^ *thi*-1 *gyr*A96 *rel*A1 | (Woodcock et al., 1989) |
| *Plasmids* |  |  |
| pME6032 | Tet^R^, P_K_, 9.8 kb pVS1 derived shuttle vector | (Heeb et al., 2000) |
| pME1114/3130/4858/5127/  5608/5698/6074 | pME6032 with SBW25 *PFLU_1114/3130/4858/5127/*  *5608/5698/6074* as *EcoRI-KpnI* fragments | This study |
| pME3087 | Tet^R^, suicide vector; ColE1-replicon, IncP-1, Mob | (Voisard C et al., 1994) |
| pME3087-1114/3130/4858/  5127/5608/5698/6074 | pME3087 with *PFLU_1114/3130/4858/5127/5608/*  *5698/6074* flanking regions as *EcoRI-BamHI* fragments | This study |
| pIJ-11-282 | pJP2 derivative with *luxCDABE* cassette expressed from  *nptII* promoter. Luminescent marker plasmid | (Frederix et al., 2014) |
| pALMAR3 | Insertion vector for Tet^r^ Mariner transposon | (Malone et al., 2010) |

Campilongo, R., Fung, R.K.Y., Little, R.H., Grenga, L., Trampari, E., Pepe, S., Chandra, G., Stevenson, C.E.M., Roncarati, D., and Malone, J.G. (2017). One ligand, two regulators and three binding sites: How KDPG controls primary carbon metabolism in Pseudomonas. *PLoS Genet* 13**,** e1006839.

Frederix, M., Edwards, A., Swiderska, A., Stanger, A., Karunakaran, R., Williams, A., Abbruscato, P., Sanchez-Contreras, M., Poole, P.S., and Downie, J.A. (2014). Mutation of praR in Rhizobium leguminosarum enhances root biofilms, improving nodulation competitiveness by increased expression of attachment proteins. *Mol Microbiol* 93**,** 464-478.

Heeb, S., Itoh, Y., Nishijyo, T., Schnider, U., Keel, C., Wade, J., Walsh, U., O'gara, F., and Haas, D. (2000). Small, stable shuttle vectors based on the minimal pVS1 replicon for use in gram-negative, plant-associated bacteria. *Mol Plant Microbe Interact* 13**,** 232-237.

Malone, J.G., Jaeger, T., Spangler, C., Ritz, D., Spang, A., Arrieumerlou, C., Kaever, V., Landmann, R., and Jenal, U. (2010). YfiBNR mediates cyclic di-GMP dependent small colony variant formation and persistence in Pseudomonas aeruginosa. *PLoS Pathog* 6**,** e1000804.

Rainey, P.B., and Bailey, M.J. (1996). Physical and genetic map of the Pseudomonas fluorescens SBW25 chromosome. *Mol Microbiol* 19**,** 521-533.

Voisard C, Bull Ct, Keel C, Laville J, Maurhofer M, and U, S. (1994). Biocontrol of root diseases by Pseudomonas fluorescens CHA0: current concepts and experimental approaches. *O'Gara F, Dowling DN, Boesten B (eds). Molecular Ecology of Rhizosphere Microorganisms.* **,** 67-89.

Woodcock, D.M., Crowther, P.J., Doherty, J., Jefferson, S., Decruz, E., Noyer-Weidner, M., Smith, S.S., Michael, M.Z., and Graham, M.W. (1989). Quantitative evaluation of Escherichia coli host strains for tolerance to cytosine methylation in plasmid and phage recombinants. *Nucleic Acids Res* 17**,** 3469-3478.
