# Supplementary material for: Differential Regulation of Genes for Cyclic-di-GMP Metabolism Orchestrates Adaptive Changes During Rhizosphere Colonization by Pseudomonas fluorescens
Source: Front Microbiol. 2019 May 16;10:1089. doi: 10.3389/fmicb.2019.01089 (PMC6531821; doi:10.3389/fmicb.2019.01089)
Supplement: Supplementary file 2 [file Table_2.DOCX]

| Table S2. Primers used in this study. | | | |
| --- | --- | --- | --- |
|  |  | |  |
| Number/Name | Sequence (5’→3’) | Description | |
| 1/ 5592RT-PCR1 | CAACGAAGTAGACGAAAGCTG | RT-qPCR forward probe for *PFLU5592*. Control primer | |
| 2/ 5592RT-PCR2 | GACGGTTGATGTCCTTGATCTC | RT-qPCR reverse probe for *PFLU5592*. Control primer | |
| 3/ 1114RT-PCR1 | TTTACCCAATGACAGCGGCAC | RT-qPCR forward probe for *PFLU1114* | |
| 4/ 1114RT-PCR2 | GAATGAAATCCACGGGGCTGAC | RT-qPCR reverse probe for *PFLU1114* | |
| 5/ 3130RT-PCR1 | AAACACCCAGCACACCATC | RT-qPCR forward probe for *PFLU3130* | |
| 6/ 3130RT-PCR2 | GCCTCTTTCAAAAACGCCAAC | RT-qPCR reverse probe for *PFLU3130* | |
| 7/ 5127RT-PCR1 | AACGACGCAATCAATCCC | RT-qPCR forward probe for *PFLU5127* | |
| 8/ 5127RT-PCR2 | CACATTCTTCCAGCAGCAC | RT-qPCR reverse probe for *PFLU5127* | |
| 9/ 5608RT-PCR1 | ACATCGCCTACTTCCTGCTC | RT-qPCR forward probe for *PFLU5608* | |
| 10/ 5608RT-PCR2 | AATCTCCGCCAACCAGTTC | RT-qPCR reverse probe for *PFLU5608* | |
| 11/ 5698RT-PCR1 | CACGGCGATTCAAAACAAG | RT-qPCR forward probe for *PFLU5698* | |
| 12/ 5698RT-PCR2 | CCAGCAATGGCAAGAACAG | RT-qPCR reverse probe for *PFLU5698* | |
| 13/ 6074RT-PCR1 | CATTGGGTGTGGATCGAAGAC | RT-qPCR forward probe for *PFLU6074* | |
| 14/ 6074RT-PCR2 | GAGGATCACAATGCCCTCGCTG | RT-qPCR reverse probe for *PFLU6074* | |
| 15/ PFLU1114UPF | CGGGATCCTTCCAGGGCCTGCTGCAG | *PFLU1114* deletion; forward primer, upstream | |
| 16/ PFLU1114UPR | CGTCTAGACGGGGTCATACGGCGCAC | *PFLU1114* deletion; reverse primer, upstream | |
| 17/ PFLU1114DNF | CGTCTAGACTGATGCCCGATCAATAG | *PFLU1114* deletion; forward primer, downstream | |
| 18/ PFLU1114DNR | CGGAATTCCTGGATCGTGAGGATATC | *PFLU1114* deletion; reverse primer, downstream | |
| 19/ PFLU3130UPF | CGGAATTCCAGTTCCTCGAGTTGAGC | *PFLU3130* deletion; forward primer, upstream | |
| 20/ PFLU3130UPR | CGTCTAGACATGGAGAGGACTCATATG | *PFLU3130* deletion; reverse primer, upstream | |
| 21/ PFLU3130DNF | CGTCTAGATTGCCGGTGGAAACCTTG | *PFLU3130* deletion; forward primer, downstream | |
| 22/ PFLU3130DNR | CGGGATCCAACAGCAGGGCGAAGATC | *PFLU3130* deletion; reverse primer, downstream | |
| 23/ PFLU4858UPF | CGGGATCCGTCCAAAGTATGTGGAAG | *PFLU4858* deletion; forward primer, upstream | |
| 24/ PFLU4858UPR | CGTCTAGAGTTCTTGAGTTCCAGCTTC | *PFLU4858* deletion; reverse primer, upstream | |
| 25/ PFLU4858DNF | CGTCTAGAAATAACGCAGCGATTCTC | *PFLU4858* deletion; forward primer, downstream | |
| 26/ PFLU4858DNR | CGGAATTCGTTACCCAGGGCATGTTC | *PFLU4858* deletion; reverse primer, downstream | |
| 27/ PFLU5127UPF | CGGGATCCCAGATCGGATGGCCGCAG | *PFLU5127* deletion; forward primer, upstream | |
| 28/ PFLU5127UPR | CGTCTAGAGTGTTTCCCGATATCCAAG | *PFLU5127* deletion; reverse primer, upstream | |
| 29/ PFLU5127DNF | CGTCTAGAAAGGACGGGGTGTCGGTG | *PFLU5127* deletion; forward primer, downstream | |
| 30/ PFLU5127DNR | CGGAATTCCGTACCAATGGCATGAAG | *PFLU5127* deletion; reverse primer, downstream | |
| 31/ PFLU5608UPF | CGGGATCCGGGGCGAGTTGACCATG | *PFLU5608*deletion; forward primer, upstream | |
| 32/ PFLU5608UPR | CGTCTAGAGCGCGACAAGGGTAAAAC | *PFLU5608* deletion; reverse primer, upstream | |
| 33/ PFLU5608DNF | CGTCTAGAGCTGCCGGTTGAGTGATG | *PFLU5608* deletion; forward primer, downstream | |
| 34/ PFLU5608DNR | CGGAATTCCATGGTGCCGGTCCACTC | *PFLU5608* deletion; reverse primer, downstream | |
| 35/ PFLU5608UPF | CGGAATTCGTAGAGGGCACCTGATTG | *PFLU5698*deletion; forward primer, upstream | |
| 36/ PFLU5608UPR | CGTCTAGATAGGTGTGGAGCGCAATC | *PFLU5698* deletion; reverse primer, upstream | |
| 37/ PFLU5608DNF | CGTCTAGATCACGCGCCCGCGTGCGTTG | *PFLU5698* deletion; forward primer, downstream | |
| 38/ PFLU5608DNR | CGGGATCCCGGCGGTAGAAGCTGCTC | *PFLU5698* deletion; reverse primer, downstream | |
| 39/ PFLU6074UPF | CGGGATCCCAGCGCTGTTCGTCGAG | *PFLU6074* deletion; forward primer, upstream | |
| 40/ PFLU6074UPR | CGTCTAGATCGACGGAGCATACACAAG | *PFLU6074* deletion; reverse primer, upstream | |
| 41/ PFLU6074DNF | CGTCTAGAAATGCAGATTTTCCTGAG | *PFLU6074* deletion; forward primer, downstream | |
| 42/ FLU6074DNR | CGGAATTCATGTGCGGGTCACTGTAG | *PFLU6074* deletion; reverse primer, downstream | |
| 43/ PFLU0179UpF3 | CGGGATCCGAAATCTCAAACGAATCC | *PFLU0179* deletion; forward primer, upstream | |
| 44/ PFLU0179UPR | CGTCTAGAGCGGCGTTTGATTTCAGTG | *PFLU0179* deletion; reverse primer, upstream | |
| 45/ PFLU0179DNF | CGTCTAGACAGGGCCAATTTGGAAAG | *PFLU0179* deletion; forward primer, downstream | |
| 46/ PFLU0179DNR | CGGAATTCGATCGGTCAATGTGTGGTC | *PFLU0179* deletion; reverse primer, downstream | |
| 47/ HexRfw | GATTCCCAGCTGTTCCGCCAGCAGGCC  TTTATC | HexR binding site mutation; forward primer | |
| 48/ HexRrv | GATAAAGGCCTGCTGGCGGAACAGCTG  GGAATC | HexR binding site mutation; reverse primer | |
| 49/ PFLU0180upN2 | CGGGATCCGAAATCTCAAACGAATCC | Forward primer downstream of HexR binding site | |
| 50/ HexRUpRnew | AAGGCCTGCTGGCGGAACAGCTGGGAATC | Forward primer upstream of HexR binding site | |
| 51/ PFLU1114-OEFor | CCTGAATTCGTGCGCCGTATGACC | PFLU1114 overexpression; forward primer | |
| 52/ PFLU1114-OERev | TAGGTACCTCGGCTATTGATCG | PFLU1114 overexpression; reverse primer | |
| 53/ 3130for | CGGAATTCATGGCCAGCCAACCCGCGAC | PFLU3130 overexpression; forward primer | |
| 54/ 3130rev | CGGGTACCTCACGGCCCCTCTAAG | PFLU3130 overexpression; reverse primer | |
| 55/ PFLU4858O-EFor | CTTGAATTCTTGAAGCTGGAACTCAAG | PFLU4858 overexpression; forward primer | |
| 56/ PFLU4858-OERev | TCCTCGAGCACGTCAGAGAATCGCTGC | PFLU4858 overexpression; reverse primer | |
| 57/ PFLU5127-OEFor | CTAGAATTCTTGGATATCGGGAAACACG | PFLU5127 overexpression; forward primer | |
| 58/ FLU5127-OERev | ATGGTACCGGGTGTTACACCGACACC | PFLU5127 overexpression; reverse primer | |
| 59/ PFLU5608-OEFor | CCAGAATTCTTGCGGGTCGGAGTTTTACC | PFLU5608 overexpression; forward primer | |
| 60/ PFLU5608-OERev | GTAGATCTTCACTCAACCGGCAGC | PFLU5608 overexpression; reverse primer | |
| 61/ PFLU5698-OEFor | CTACAATTGATGGATTGCGCTCCACAC | PFLU5698 overexpression; forward primer | |
| 62/ PFLU5698-OERev | GCGGTACCACGAATTCAACGCACG | PFLU5698 overexpression; reverse primer | |
| 63/ PFLU6074-OEFor | TGGAGCTCTTGTGTATGCTCCGTCGAC | PFLU6074 overexpression; forward primer | |
| 64/ PFLU6074-OERev | GCGGTACCGCATCACTCAGGAAAATC | PFLU6074 overexpression; reverse primer | |
| 65/ Arb-PCR | CGCAAACCAACCCTTGGCAG | Locating transposition insertion site | |
| 66/ Arb1 | GGCCAGCGAGCTAACGAGAC | Locating transposition insertion site | |
| 67/ Arb1b | GGCCAGCGAGCTAACGAGACNNNNGATAT | Locating transposition insertion site | |
| 68/ Almar3-seq | ACATATCCATCGCGTCCGCC | Locating transposition insertion site | |
