# Supplementary figures and images for: Differential Regulation of Genes for Cyclic-di-GMP Metabolism Orchestrates Adaptive Changes During Rhizosphere Colonization by Pseudomonas fluorescens
Source: Front Microbiol. 2019 May 16;10:1089. doi: 10.3389/fmicb.2019.01089 (PMC6531821; doi:10.3389/fmicb.2019.01089)

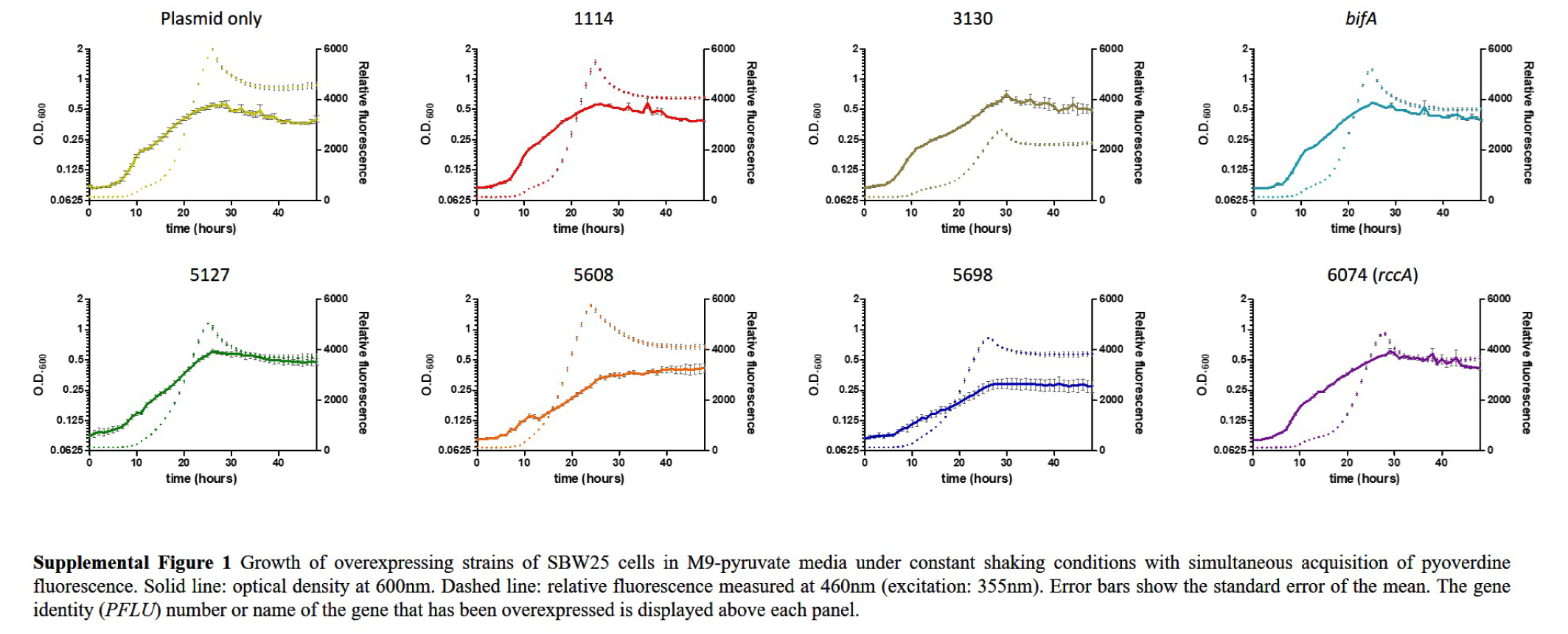

Supplement: Supplementary file 3 [file Image_1.TIF]

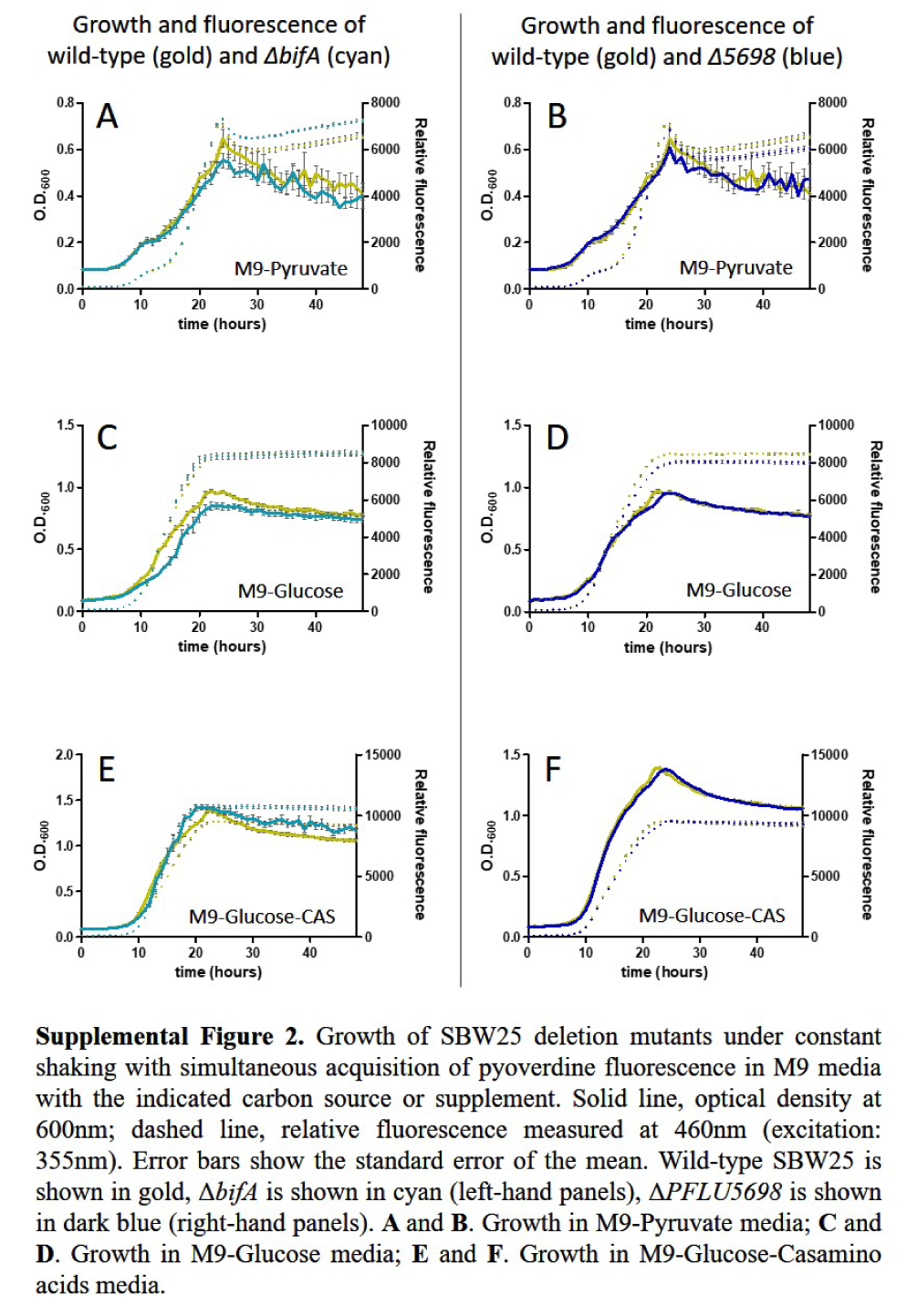

Supplement: Supplementary file 4 [file Image_2.TIF]

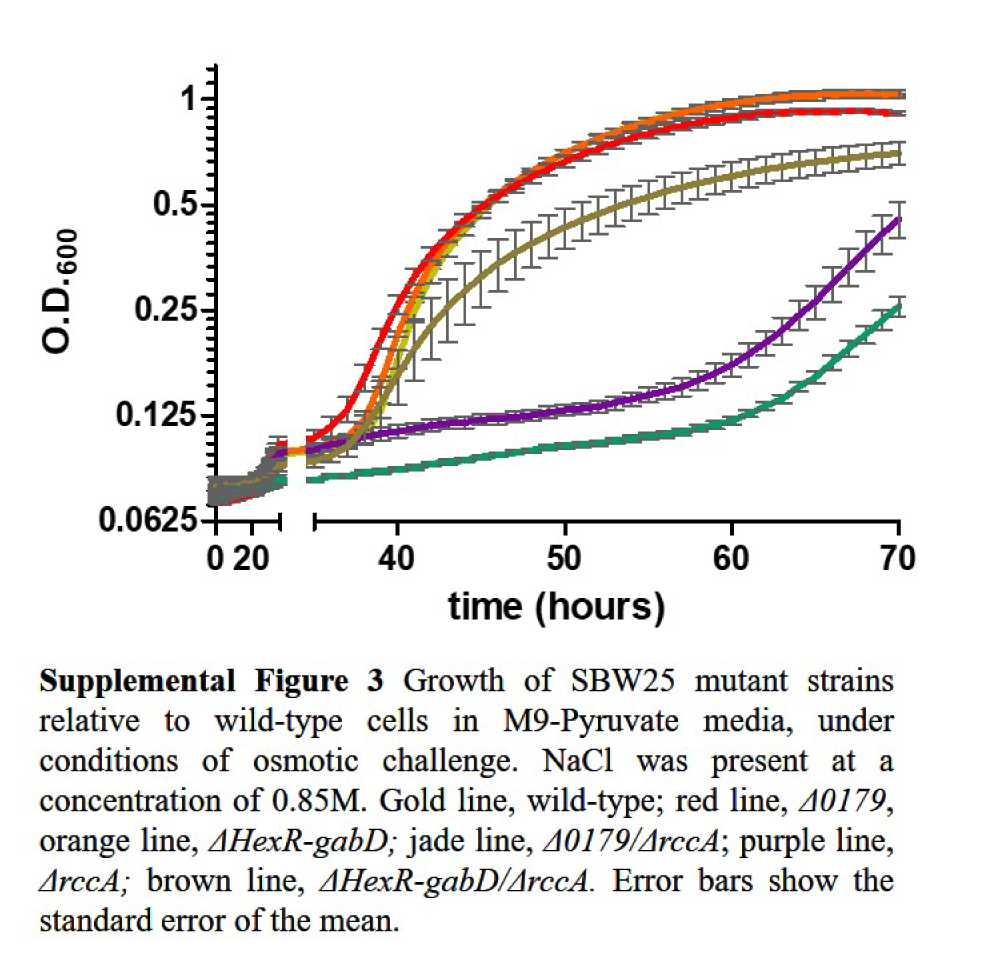

Supplement: Supplementary file 5 [file Image_3.TIF]
